# Computed Tomography Radiomics and Machine Learning for Prediction of Histology-Based Hepatic Steatosis Scores

**DOI:** 10.3390/diagnostics15182310

**Published:** 2025-09-11

**Authors:** Winston T. Chu, Hui Wang, Marcelo A. Castro, Venkatesh Mani, C. Paul Morris, Thomas C. Friedrich, David H. O’Connor, Courtney L. Finch, Ji Hyun Lee, Philip J. Sayre, Gabriella Worwa, Anya Crane, Jens H. Kuhn, Ian Crozier, Jeffrey Solomon, Claudia Calcagno

**Affiliations:** 1 Integrated Research Facility at Fort Detrick, Division of Clinical Research, National Institute of Allergy and Infectious Diseases, National Institutes of Health, B-8200 Research Plaza, Frederick, MD 21702, USA; winston.chu@nih.gov (W.T.C.); marcelo.castro@nih.gov (M.A.C.); venky.mani@nih.gov (V.M.); christopher.morris@nih.gov (C.P.M.); jihyun.lee@nih.gov (J.H.L.); gabriella.worwa@nih.gov (G.W.); anya.crane@nih.gov (A.C.); kuhnjens@mail.nih.gov (J.H.K.); 2 Center for Infectious Disease Imaging, Radiology and Imaging Sciences, Clinical Center, National Institutes of Health, 10 Center Drive, Bethesda, MD 20892, USA; 3 Department of Pathobiological Sciences, School of Veterinary Medicine, University of Wisconsin-Madison, 2015 Linden Drive, Madison, WI 23706, USA; thomasf@primate.wisc.edu; 4 Department of Pathology and Laboratory Medicine, University of Wisconsin-Madison, 1685 Highland Avenue, Madison, WI 53705, USA; dhoconno@wisc.edu; 5 Clinical Monitoring Research Program Directorate, Frederick National Laboratory for Cancer Research, 5705 Industry Lane, Frederick, MD 21704, USA; ian.crozier@nih.gov (I.C.); jeffrey.solomon@nih.gov (J.S.)

**Keywords:** computed tomography, radiomics, machine learning, hepatic steatosis, nonhuman primates

## Abstract

**Background/Objective:** Computed tomography (CT) can be used to non-invasively assess the health of the liver; however, radiologist evaluation and simple thresholding alone are insufficient for diagnosis of hepatic steatosis, necessitating biopsies. This study explored CT radiomics and machine learning to enable non-invasive, objective, and quantitative prediction of steatosis severity across the macaque liver. **Methods:** In this retrospective study, CT images of 42 crab-eating macaques (age [yr] = 6.1 ± 1.7; sex [male/female] = 26/16) with varying degrees of hepatic steatosis were analyzed, and the results were compared to histology-based steatosis scores of livers from the same animals. After extracting radiomic features, a thorough array of statistical analyses, feature selection techniques, and machine learning models were applied to identify a distinct radiomic signature of histologically defined hepatic steatosis. **Results:** We identified 12 radiomic features that correlated with steatosis scores, and hierarchical clustering based on radiomic attributes alone revealed clusters roughly aligning with steatosis severity groups. The k-nearest neighbors model architecture best predicted histopathologic steatosis scores in both classification and regression tasks (area under the receiver operating characteristic curve [AUC ROC] = 0.89 ± 0.09; root-mean-square error [RMSE] = 0.60 ± 0.10). Feature analyses identified seven key radiomic features (six first-order features and one gray-level co-occurrence matrix feature) that were most important when predicting steatosis. **Conclusions:** We identified a CT radiomic signature of steatosis and demonstrated that histology-based steatosis scores can be predicted non-invasively and objectively using machine learning and CT radiomics as a potential alternative to invasive core biopsies. Given the strong similarities in liver structure, liver function, and hepatic steatosis pathophysiology between macaques and humans, these findings have the potential to translate to humans.

## 1. Introduction

Hepatic steatosis is an early hallmark of various liver diseases. In nonalcoholic and alcoholic fatty liver disease (NAFLD and AFLD), chronic hepatic steatosis is a precursor to the development of inflammation (steatohepatitis), fibrosis, and cirrhosis, which contribute to liver failure [1]. Hepatic steatosis is a risk factor for severe and fatal outcomes after infection with viruses such as severe acute respiratory syndrome coronavirus 2 (SARS-CoV-2) [2,3,4]. Further research and more advanced tools are needed to identify and quantify hepatic steatosis to characterize its pathophysiologic role in the progression of liver diseases and its contribution to clinical outcomes.

A definitive diagnosis of hepatic steatosis and steatohepatitis currently requires a liver core biopsy, sample assessment by a pathologist, and characterization using histopathologic feature scoring criteria. For example, the widely used NAFLD activity score (NAS) system [5] incorporates an unweighted graded assessment of steatosis, hepatocyte ballooning, and lobular inflammation to assess the severity of liver disease. However, sparse sampling of a large organ may produce over- or under-represented histopathologic features (i.e., sampling bias) that are not uniform across the parenchyma, such as steatosis in NAFLD [6]. Furthermore, visual assessment is inherently subjective, resulting in poor to moderate reliability among scorers (intraclass correlation coefficients of 0.49–0.72) using the NAS [7,8].

The weaknesses of core biopsies not only affect clinical diagnosis of NAFLD but also impact research into the pathophysiology of NAFLD. Animal models of NAFLD enable a more thorough assessment of liver tissue because multiple samples from the same liver can be assessed histologically, reducing sampling bias. Crab-eating macaques serve as a particularly effective model of human NAFLD, given their strong similarities in liver structure and function, NAFLD pathogenesis, and responses to therapeutic NAFLD treatments [9,10,11,12,13].

Medical imaging methods, such as computed tomography (CT), may enable a more comprehensive evaluation of the liver compared to biopsies. Although CT imaging can be used to non-invasively evaluate the whole liver in vivo, qualitative radiologic analyses of liver CT images are not yet considered specific enough to eliminate the need for invasive biopsies [14]. Furthermore, no consensus has been reached on a simple CT attenuation threshold for diagnosing hepatic steatosis [15]. Application of explainable artificial intelligence and mathematically discrete image analysis methods, such as radiomics, may generate quantitative and disease-specific radiological CT signatures, thereby improving the clinical value of imaging-based assessment of liver disease.

Radiomics is an image analysis method used to automatically quantify patterns of intensities and textures in medical images, many of which are not perceivable by the unaided human eye [16]. It extracts discrete mathematically defined and scalar features that can be used by classical machine learning models to predict specific disease hallmarks.

**Figure 1:**
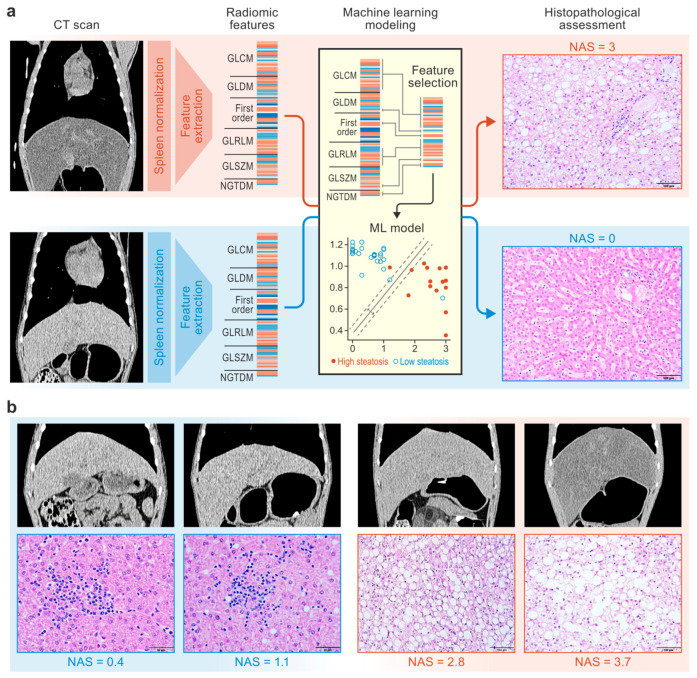
Study design. CT scans and liver tissue samples from macaques were used to train machine learning models to predict histopathologic scores of steatosis severity (**a**). Representative CT scans (window = 270 HU, level = 15 HU, gamma = 1) and hematoxylin-and-eosin-stained liver tissue images from macaques with NAS scores ranging from 0 to 3.7 are shown (**b**). CT, computed tomography; GLCM, gray-level co-occurrence matrix; GLDM, gray-level dependence matrix; GLRLM, gray-level run length matrix; GLSZM, gray-level size zone matrix; ML, machine learning; NAFLD, nonalcoholic fatty liver disease; NAS, NAFLD activity score; NGTDM, neighboring gray-tone difference matrix.

In this study, we hypothesized that CT radiomics and machine learning could identify non-invasive and disease-specific imaging signatures of histologically defined liver steatosis and therefore aid in the objective and explainable prediction of this important hallmark of liver disease. To test this hypothesis, we leveraged a dataset of 42 CT scans and liver histology slides collected from crab-eating macaques with varying degrees of steatosis (as confirmed by the distribution of liver histopathology scores obtained from necropsy samples). After extracting radiomic features, an array of feature selection techniques were performed and machine learning models were trained and tested on the dataset (Figure 1). The highest-performing model was further analyzed, and a radiomic signature of histologically defined steatosis was identified. Our findings shed light on the connections between histopathological and specific CT radiomic features of liver disease and suggest that histology-based hepatic steatosis severity scores may be predicted using non-invasive CT radiomics and machine learning. Our study builds towards research as well as clinical applications of CT imaging to non-invasively characterize liver disease, predict disease progression, and predict responses to therapies.

## 2. Materials and Methods

### 2.1. Study Overview

A dataset was built with terminal data related to 42 crab-eating macaques (*Macaca fascicularis* Raffles, 1821) from multiple SARS-CoV-2 exposure studies that were performed in our facility. The inclusion criteria for this retrospective study were (1) CT image acquisition using the same scanner and identical parameter settings and (2) liver tissue collection within 24 h of CT imaging. (Note: Images from 6 of the 42 animals were used in a previous publication with a different scope [17]). Data were collected from virus-exposed and unexposed male and female macaques of various ages with three geographic origins (Table 1) and included two cohorts distinguished by diet. Specifically, for a minimum of 21 months before imaging, 15 of the 42 macaques were fed a high-fat high-fructose (atherogenic) diet to induce high cholesterol concentrations in their plasma as part of a well-characterized model of atherosclerosis [18,19]. The other 27 macaques were fed a standard (non-atherogenic) monkey diet (Table S1). CT imaging was performed in vivo, and liver parenchyma radiomic features were extracted using standardized methods [16]. Liver tissues collected during necropsies were assessed by a board-certified pathologist, who confirmed steatosis and assigned a score according to NAS criteria [5]. NAS steatosis sub-score data were binarized for hierarchical clustering and machine learning classification analyses. Machine learning models were then trained to predict histology-based steatosis severity scores using CT radiomic data (Figure 1).

### 2.2. Histopathology

All macaques were euthanized within 24 h after terminal CT imaging. Liver specimens were collected by a pathologist during necropsy. Tissues were formalin-fixed, paraffin-embedded, sliced at a thickness of 4 µm, and stained with hematoxylin and eosin (H&E). Subsequently, a board-certified veterinary anatomic pathologist examined the slides. Each liver section was assessed, with 10 fields examined at a total magnification of 100x. Based on involvement in the parenchyma, steatosis was scored as follows: 0 (<5%), 1 (5–33%), 2 (33–66%), and 3 (>66%). The average score across all fields was recorded as the NAS steatosis sub-score for each macaque. Assessments were also made for hepatic inflammation and ballooning activity. The NAS was calculated by combining the steatosis, inflammation, and ballooning sub-scores. All grading and staging were performed according to the standard Kleiner Classification [5].

For binary analyses of steatosis severity, the midpoint (1.5) of the NAS steatosis sub-score range (0–3) was used as a threshold to form low- and high-steatosis groups.

### 2.3. CT Imaging

#### 2.3.1. Data Acquisition

Images were acquired using the 16-slice CT component of a Gemini TF 16 scanner (Philips Healthcare, Cleveland, OH, USA). The macaques were positioned in a supine head-out/feet-in orientation and connected to a ventilator to facilitate 15–20 s breath holds with a maintained pressure of 150 mmH_2_O. Vital signs were monitored throughout the procedure. The parameter settings included an ultra-high resolution, 140 kVp, 300 mAs, a thickness of 1 mm, an increment of 0.5 mm, a pitch of 0.688 mm, 16 × 0.75 collimation, and 0.75 s per rotation. For image reconstruction, a bone-enhanced D kernel was used to increase the sharpness, utilizing a matrix size of 512 × 512 × 343 with a field of view of 180 × 180 × 171.5 mm, resulting in a pixel size of 0.35 × 0.35 × 0.5 mm.

#### 2.3.2. Image Preprocessing and Region-of-Interest Segmentation

The spleen-normalized liver Hounsfield unit (HU) is often used as a criterion to diagnose liver diseases, such as steatosis [20]. Additionally, spleen normalization improves radiomic feature reliability in the liver [17]. Therefore, D-kernel reconstructed images were normalized by the mean spleen HU value. Spleen segmentation masks were generated using a previously described feature pyramid network, which is a type of convolutional neural network [21,22]. Liver-to-spleen attenuation normalization was computed using a custom-built workflow in MIM version 7.1.6 (MIM Software, Cleveland, OH, USA) in which each pixel was divided by the mean attenuation computed from the segmented spleen (producing a unitless image).

For downstream calculation of liver radiomic features, six three-dimensional spherical regions of interest (ROIs) were manually placed within the liver parenchyma, each with a radius of 10 mm. Four were placed in the right lobe (i.e., right superior, right mid-center, right mid-lateral, and right inferior regions) and two were placed in the left lobe (i.e., left mid and left lateral regions). The ROIs were placed manually by a single experienced annotator, ensuring no inter-observer variability. Size differences in small ROIs have been shown to produce false differences in CT radiomic features [23]; therefore, standardized ROIs may be superior for radiomic analyses compared to variably sized ROIs.

#### 2.3.3. Radiomic Feature Extraction

Spleen-normalized D-kernel liver CT scans were discretized with a bin width of 0.02 (unitless), resulting in approximately 150 bins per image. Compared to a fixed number of bins, a fixed bin width improves the comparability of textural features in positron emission tomography [24]. Radiomic feature extraction was performed using PyRadiomics version 2.2.0 [16] and MIM. A full mathematical definition of each radiomic feature can be found on the PyRadiomics website (https://pyradiomics.readthedocs.io/en/2.2.0/features.html (accessed on 3 April 2025)), and these definitions are largely in compliance with the feature definitions described by the Imaging Biomarkers Standardization Initiative [25]. A total of 94 features (19 first-order [intensity] features and 75 second-order [textural] features) were extracted from each of the six ROIs within the normalized D-kernel CT scans, and the average radiomic feature values across the six ROIs were calculated. Table S2 provides a full list of the radiomic features examined in this study.

### 2.4. Statistical Analysis

Statistical analyses were used to characterize the data and identify trends that would inform the machine learning analysis. Welch’s *t*-tests and Chi-squared tests were used to characterize the differences in demographic attributes between the low- and high-steatosis groups, and Bonferroni-corrected *p*-values were calculated. For each radiomic feature, the Pearson’s correlations with the histopathology features were calculated to identify significant linear relationships after Bonferroni correction. Histogram plots were generated to visualize the distributions of the NAS sub-scores. A hierarchical clustering map was generated to examine the relationships among the subjects and among the radiomic features in an unsupervised manner. Finally, a correlation map was generated to visualize the correlations among the radiomic features and determine the degree of collinearity in the dataset.

In summary, the statistical analyses were based on 42 independent biological data points (i.e., 42 CT scans and H&E slides from 42 macaques). Six fields from the liver of each CT scan and ten fields from each liver section were averaged to generate the radiomic values and histopathologic scores, respectively. The performances of the machine learning models were assessed with 20 iterations of cross-validation. This was a retrospective study; therefore, the sample size was determined based on the available data. Randomization and blinding did not occur.

### 2.5. Machine Learning Analysis

#### 2.5.1. Computing Resources and Libraries

This study used the Office of Cyber Infrastructure and Computational Biology (OCICB) High Performance Computing (HPC) cluster at the National Institute of Allergy and Infectious Diseases (NIAID), Bethesda, MD, USA. Statistical analyses, machine learning analyses, and visualization were performed using the NumPy (version 1.26.4; https://numpy.org (accessed on 3 April 2025)), Pandas (version 2.2.2; https://pandas.pydata.org (accessed on 3 April 2025)), Scikit-learn (version 1.4.2; https://scikit-learn.org (accessed on 3 April 2025)), Matplotlib (version 3.8.4; https://matplotlib.org (accessed on 3 April 2025)), and Seaborn (version 0.13.2; https://seaborn.pydata.org (accessed on 3 April 2025)) Python (version 3.12.3) libraries.

#### 2.5.2. Model Development

Machine learning models were trained with liver CT radiomic features to predict histology-based hepatic steatosis scores. Specifically, binary classification models were trained to predict NAS steatosis sub-scores above 1.5, and regression models were trained to predict unmodified NAS steatosis sub-scores. Eight model architectures were examined for classification and regression tasks. These included linear models (i.e., logistic/linear regression and a support vector machine), ensemble tree-based models (i.e., random forest, gradient boosting, and AdaBoost), decision trees, k-nearest neighbors (kNN), and a multi-layer perceptron. These models were implemented using the Scikit-learn Python package with the default parameters [26].

For the classification task, 10 feature selection techniques were examined:

1. Univariate f-test feature selection (0.05 *p*-value threshold) [26];
2. Univariate f-test feature selection (0.005 *p*-value threshold);
3. Least absolute shrinkage and selection operator (LASSO) regression [26,27];
4. Elastic Net regression [26,28];
5. Minimum redundancy, maximum relevance (mRMR) feature selection (top 25% of features) [29];
6. mRMR feature selection (top 50% of features);
7. mRMR feature selection (top 75% of features);
8. mRMR-permute [30];
9. Repeatable feature criteria [17,31];
10. No feature selection.

These techniques were chosen to compare state-of-the-art techniques (i.e., f-test, LASSO, Elastic Net, and mRMR) with emerging techniques (i.e., mRMR-permute and repeatable feature criteria). The univariate f-test feature selection methods chose features that were statistically significantly different between the steatosis groups but did not consider the interactions among the features. The f-test *p*-value thresholds of 0.05 and 0.005 were examined to test two common levels of statistical stringency. The LASSO regression feature selection method uses ordinary least squares regression with an L1 penalty term. It prioritized features that varied linearly in the steatosis group while eliminating features that were redundant or poorly correlated with the steatosis group [27]. The Elastic Net regression feature selection method also uses ordinary least squares regression but with both L1 and L2 penalty terms. The addition of the L2 penalty term stabilizes coefficient optimization and allows Elastic Net to keep groups of correlated features, rather than arbitrarily choosing one from a group of correlated features [28]. The mRMR technique scored the features by maximizing the mutual information between the steatosis group and the features while minimizing the mutual information between the features [29]. The mRMR technique does not recommend a feature set size or a score threshold. Users of mRMR must choose a target feature set size, and, in this study, we examined feature sets that consisted of the top 50 and 75% of features to ensure that all relevant features were included. The mRMR-permute method was developed to eliminate the bias associated with choosing a feature set size or score threshold.

mRMR-permute is an emerging technique that combines mRMR and permutation testing for fully automated and unbiased feature selection. The mRMR-permute technique uses permutation testing to derive a threshold for the mRMR score so that the features can be thresholded at a *p*-value rather than with an arbitrarily chosen feature set size [30]. In addition to data-driven feature selection techniques, a feature set derived from repeatability criteria was examined [17]. Wang and colleagues determined which radiomic features are repeatable in the liver by examining radiomic feature variability across repeat CT scans. The exhaustive comparison of emerging feature selection techniques (such as mRMR-permute and repeatability criteria) with state-of-the-art feature selection techniques and their use with a wide array of state-of-the-art machine learning architectures represents a unique and significant technical contribution of this work.

For regression modeling, the mRMR and mRMR-permute feature selection techniques were not considered because they cannot be easily adapted for the task of regression. Thus, 6 of the 10 feature selection techniques were examined in the regression task (i.e., univariate f-test feature selection [0.05 *p*-value threshold], univariate f-test feature selection [0.005 *p*-value threshold], LASSO, Elastic Net, repeatable feature criteria [17,31], and no feature selection).

For both classification and regression model training, each combination of feature selection techniques and models was evaluated using 20 iterations of cross-validation (CV), with 70% used for training and 30% used for testing. Feature selection was re-calculated within each CV iteration, preventing information leakage across the training and test sets and leading to more accurate estimates of model generalizability.

The classification models and feature selection techniques were compared using the average Matthew’s correlation coefficient (MCC) across the 20 CV iterations. This metric is similar to accuracy; however, it is known to better account for class imbalances [32]. The regression models and feature selection techniques were compared using the average root-mean-square error across the 20 CV iterations.

#### 2.5.3. Model Performance and Feature Analysis

The performance of the top classification model was further characterized using the receiver operating characteristic (ROC) curve, an aggregate confusion matrix, precision, sensitivity, and specificity. The performance of the top regression model was demonstrated with a Bland–Altman plot. Because feature selection was re-computed for every CV iteration, 20 CV iterations resulted in 20 feature sets. To identify the top features across the 20 feature sets, the occurrence of each feature in a feature set was plotted; we identified the most important features as those selected in more than half of the CV iterations.

## 3. Results

### 3.1. Data Characterization

**Figure 2:**
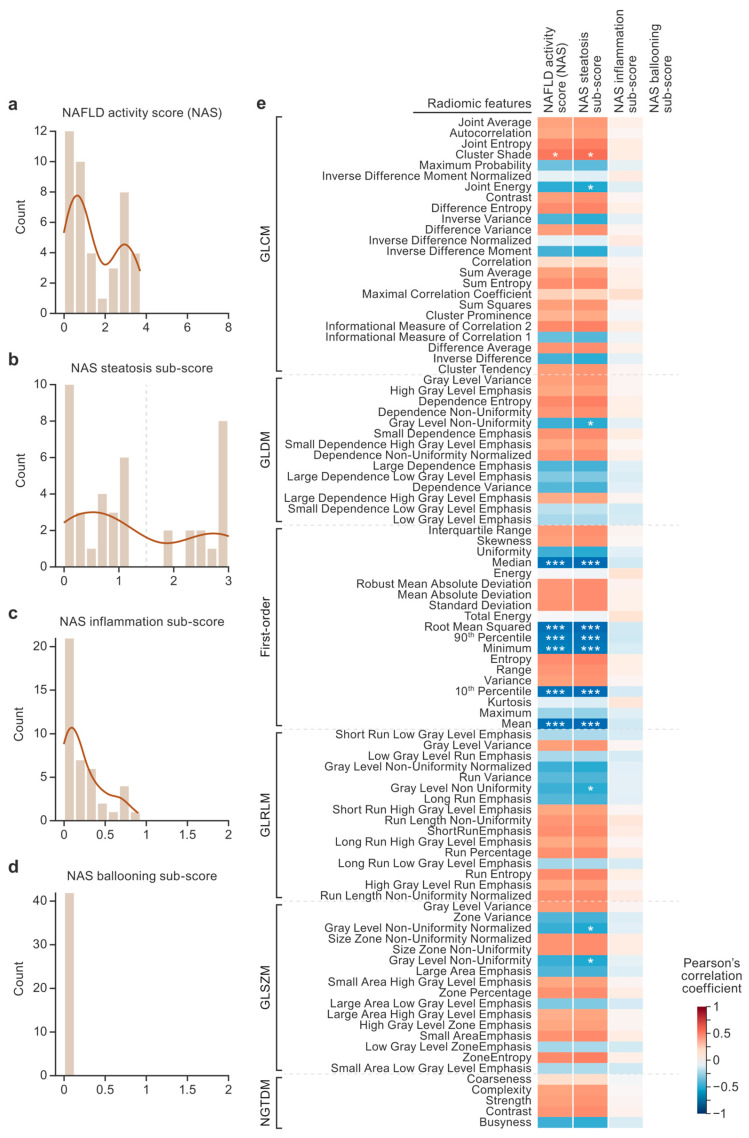
Nonalcoholic fatty liver disease (NAFLD) activity score (NAS) distributions and correlations with radiomics. The histogram plots show the distributions of NAS scores (**a**–**d**). An NAS steatosis sub-score threshold of 1.5 was used for the classification task and is designated with a dashed vertical line (**b**). The heat map shows the results of correlations between the radiomic features and pathologist scores (**e**). The radiomic features are listed on the y axis, and the pathologist scores are listed on the x axis. The color of each cell designates the value of the Pearson’s correlation coefficient. Asterisks designate statistical significance after Bonferroni correction (* *p*_Bonferroni_ < 0.05, *** *p*_Bonferroni_ < 0.005). GLCM, gray-level co-occurrence matrix; GLDM, gray-level dependence matrix; GLRLM, gray-level run length matrix; GLSZM, gray-level size zone matrix; NGTDM, neighboring gray-tone difference matrix.

Histograms of the NAS sub-scores show the distribution and range of severity observed in the histology (Figure 2a–d). The NAS had a range of 0–3.7 out of a maximum score of 8. The NAS steatosis sub-score was the only metric in which the data spanned the entire range of possible values (0–3). Bimodal distributions were observed in the NAS and the NAS steatosis sub-score. Mild inflammation in the absence of ballooning was observed histologically in the macaque livers. Therefore, in this dataset, NAS variance was largely driven by the NAS steatosis sub-scores. Correlations were performed between the radiomic features, NAS, and NAS sub-scores (Figure 2e). Six radiomic features showed very strong correlations with the NAS steatosis sub-scores (*p*_Bonferroni_ < 0.005), and all were first-order features (the median, root mean square, 90th percentile, minimum, 10th percentile, and mean). The average magnitudes of the correlations between the radiomic and histopathologic features were calculated for the NAS ($\left|r\right|=0.42$, moderate), steatosis sub-scores ($\left|r\right|=0.44$, moderate), and inflammation sub-scores ($\left|r\right|=0.081$, negligible).

A hierarchical cluster map was generated based on the z-scores of the radiomic data, revealing patterns across subjects (x axis) and across radiomic features (y axis) without relying on predefined labels (Figure S1). Three major subject clusters and two major radiomic feature clusters (designated by the dendrograms) were identified. The first-order features of radiomic feature clusters 1 and 2 differed in that cluster 1 contained more features related to intensity (the mean, median, 90th percentile, 10th percentile, and minimum) compared to cluster 2, which contained more features related to variance (the standard deviation, mean absolute deviation, robust mean absolute deviation, and range). There were no discernable patterns in the second-order features grouped in cluster 1 compared to cluster 2. Subject cluster 1 was composed of three animals (two low-steatosis animals and one high-steatosis animal) and was characterized by low values in radiomic feature cluster 1 and high values in radiomic feature cluster 2. Subject cluster 2 contained the opposite trend and was 95% composed of low-steatosis animals. Subject cluster 3 contained the same trend as subject cluster 1 and was 68% composed of high-steatosis animals. Thus, hierarchical clustering based on the radiomic data revealed clusters of subjects roughly aligned with low- and high-steatosis groups.

A correlation matrix was computed among the radiomic features (Figure S2) to assess the feature set for redundant (i.e., highly correlated) features. Many radiomic features were determined to be highly correlated, and this observation motivated the use of feature selection to reduce the dimensionality of the prediction task.

### 3.2. Machine Learning Model Selection

**Figure 3:**
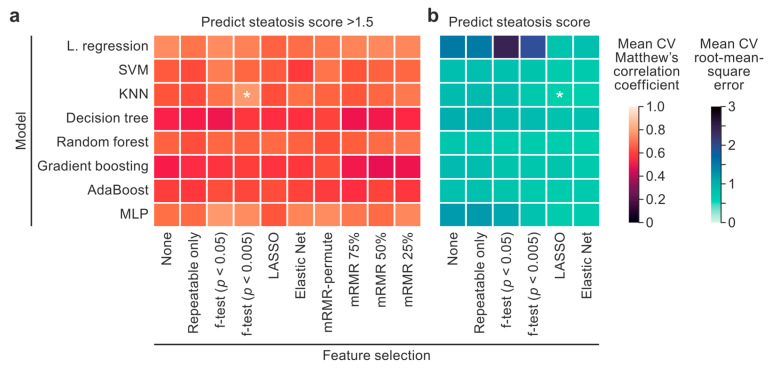
A model and feature selection technique comparison heatmap. Classification (**a**) and regression (**b**) model performances for predicting an NAS steatosis score > 1.5 and the NAS steatosis score, respectively, are plotted. The x axis lists the feature selection techniques, and the y axis lists the model architectures. The average Matthew’s correlation coefficients (MCCs) and root-mean-square errors (RMSEs) across 20 cross-validation iterations were plotted and used to compare the models. The top model and feature selection technique pairs with the highest MCCs and lowest RMSEs were analyzed further and are designated with asterisks. The color scales were set so that lighter cells designate better performance. KNN, k-nearest neighbors; LASSO, least absolute shrinkage and selector operator; L. regression, logistic/linear regression; MLP, multi-layer perceptron; mRMR, minimum redundancy, maximum relevance; SVM, support vector machine. * Top model and feature selection technique pairs.

The optimal model architecture and feature selection method for the binary classification task were chosen by comparing all combinations using the mean MCC across 20 CV iterations (Figure 3a). The top-performing model and feature selection method combination was the kNN model trained on f-test (*p* < 0.005 threshold) features (MCC = 0.792 ± 0.16). Averaged across all examined models, the f-test (*p* < 0.005 threshold) and f-test (*p* < 0.05 threshold) achieved MCC scores of 0.68 ± 0.19 and 0.66 ± 0.21 using 17 and 52 features, respectively. Averaged across all examined models, feature selection using mRMR-permute [30] achieved an MCC score of 0.66 ± 0.19 with only 11 features, demonstrating that mRMR-permute performed comparably to the top feature selection techniques with fewer features.

The optimal model architecture and feature selection method for the regression task were chosen by comparing all combinations using the mean root-mean-square error (RMSE) across 20 CV iterations (Figure 3b). A kNN model trained on LASSO features achieved the lowest RMSE of 0.60 ± 0.10.

### 3.3. Machine Learning Model Performance

**Figure 4:**
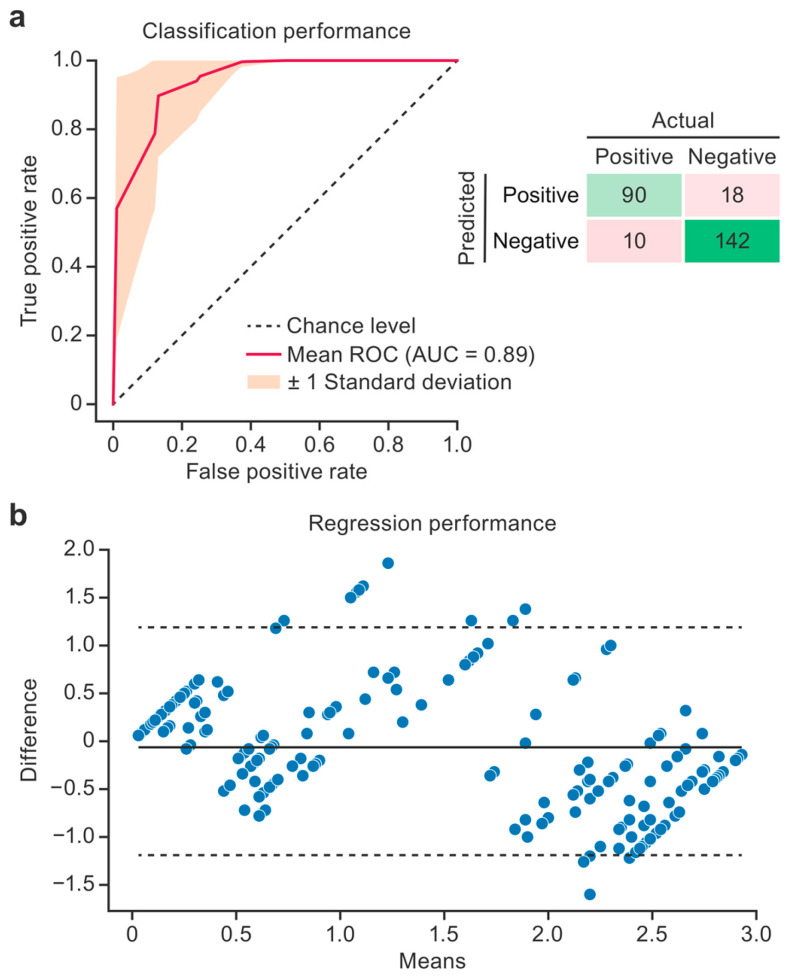
Model performance. The receiver operating characteristic (ROC) curve and aggregate confusion matrix illustrate the performance of the binary steatosis classification model across 20 cross-validation (CV) iterations (**a**). The red line gives the average model performance, the orange region designates the standard deviation (SD), and the dashed line designates the random prediction performance. The Bland–Altman plot shows the performance of the steatosis regression model (**b**). The difference between the predicted and true steatosis scores, as a function of the mean of the two scores, is plotted. The solid line designates the mean difference. The dashed lines show the 95% confidence intervals. Predictions from all 20 CV iterations are shown. AUC, area under the curve.

The top-performing classification model was the kNN model trained on features selected using the f-test criteria (*p* < 0.005), and its performance was further characterized using an ROC curve and an aggregate confusion matrix (Figure 4a). The variance in performance across 20 CV iterations is shown in the ROC plot using a ±1 standard deviation region. This model achieved an ROC area under the curve (AUC) of 0.89 ± 0.09, accuracy of 0.89 ± 0.08, precision of 0.86 ± 0.13, sensitivity of 0.90 ± 0.17, specificity of 0.89 ± 0.11, and an f1 score of 0.86 ± 0.12. No obvious trends favored sensitivity or specificity, indicating balanced performance across classes.

Bland–Altman plots (Figure 4b) show the top-performing regression model (a kNN model trained on LASSO features) by plotting the difference between the ground truth and the prediction as a function of the mean of the two scores. This model performed best for the lowest and highest steatosis severities and skewed toward higher scores in the middle of the range (1.5).

**Figure 5:**
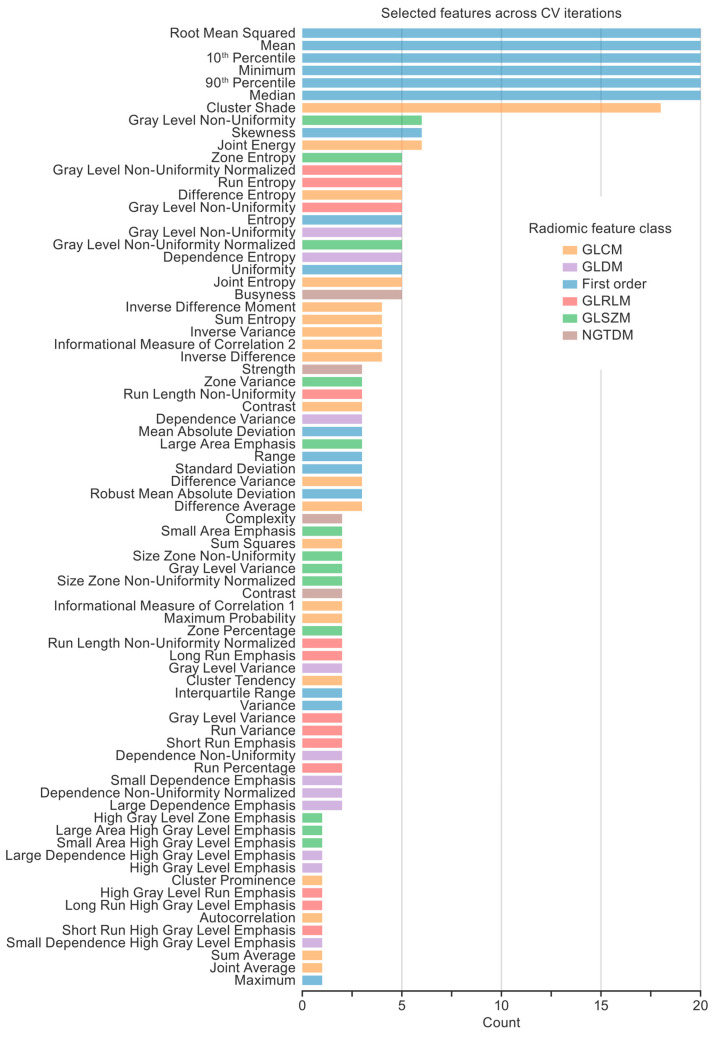
Features selected across cross-validation (CV) iterations. Twenty CV iterations resulted in 20 feature sets selected by the feature selection technique (i.e., an f-test with a 0.005 *p*-value threshold). A histogram shows the number of times each feature was selected. Seven key radiomic features (six first-order features and one gray-level co-occurrence matrix feature) were selected in more than half of the CT iterations.

Feature selection performed within the CV loops resulted in a feature set for each CV iteration. To characterize the importance of the features across the 20 CV iterations for the classification model, the frequency of each feature was plotted so that the maximum frequency of 20 indicated that a feature was selected in every CV iteration (Figure 5). Seven features were selected in more than half of the CV iterations, comprising six first-order features (the root mean square, mean, 10th percentile, minimum, 90th percentile, and median) and one gray-level co-occurrence matrix feature (GLCM; cluster shade).

## 4. Discussion

In this study, we explored the potential for AI-powered CT radiomic analysis to correlate with and predict histology-based hepatic steatosis severity scores. The relationship between CT radiomics and hepatic steatosis has previously been explored. For instance, textural features from single slices of CT scans predicted a biopsy-based diagnosis of hepatic steatosis [33]. Further, CT radiomic features predicted a radiological diagnosis of hepatic steatosis [34]. However, to our knowledge, only two studies have used CT radiomic features to predict histologic steatosis grades [35,36]. In both studies, mixtures of first- and second-order features were identified as being important for prediction. In contrast, our models prioritized simpler first-order features (i.e., the root mean square, mean, 10th percentile, minimum, 90th percentile, and median). This could be attributed to the fact that our images were spleen-normalized before the radiomic features were extracted, while the two other studies did not report spleen normalization. Spleen normalization is often performed for clinical evaluation of CT scans of the liver and has been shown to improve radiomic feature reliability in the liver [17,20]. To our knowledge, no other study has predicted histologic steatosis grades with radiomics calculated from spleen-normalized CT scans of the liver. Based on these results, we recommend spleen normalization before radiomic feature extraction to reduce the variability in liver intensities, thus making the radiomic features more uniform and interpretable.

Classical machine learning models are considered more explainable compared to deep learning models because they can use interpretable features (e.g., radiomic features), and many architectures generate feature importance metrics during model training (e.g., linear regression, a decision tree, or a linear support vector machine). In this study, the top-performing model was the kNN model; however, this model does not generate a metric of feature importance. To determine and interpret the top features, the features selected by the top-performing feature selection technique for this model were examined. Across the 20 CV iterations, seven radiomic features were selected as being the most important for predicting steatosis severity. Six of the radiomic features (i.e., the root mean square, mean, 10th percentile, minimum, 90th percentile, and median) were first-order features that describe the middle and range of the intensities in the liver. An accumulation of intracellular triglycerides and other lipids in hepatocytes is expected to lower liver attenuation. However, there is no consensus on a simple CT attenuation threshold for hepatic steatosis diagnosis [15]. The seventh radiomic feature was the GLCM cluster shade (https://pyradiomics.readthedocs.io/en/2.2.0/_modules/radiomics/glcm.html#RadiomicsGLCM.getClusterShadeFeatureValue [accessed on 3 April 2025]). The GLCM computes how often pairs of pixels (with a specific value and distance) occur in the image, and cluster shade measures the skewness and uniformity of the GLCM (higher values indicate higher skewness). GLCM cluster shade positively correlated with the NAS steatosis scores, which indicates that worse steatosis may have resulted in more clusters of low-intensity regions and, broadly, less uniformity in the intensities in the liver, which may indicate focal hepatic steatosis.

We examined an array of machine learning models and found that the kNN model performed the best for both the binary classification and regression tasks. These results aligned with our observations in the hierarchical cluster map analysis (Figure S1) that unsupervised clustering could also distinguish between the low- and high-steatosis groups. The high performance of supervised and unsupervised clustering suggests that the relationship between radiomic features and histological steatosis scores is nonlinear but has a strong local structure (i.e., subjects with similar radiomic features have similar steatosis scores). Nonlinearity and a strong local structure may explain why the kNN model outperformed models such as logistic regression and the linear support vector machine. The kNN model outperformed the decision tree, random forest, gradient-boosting, and multi-layer perceptron models, likely because it is less complex and therefore less susceptible to overfitting.

In this study, the performance of intra-subject repeatability criteria and mRMR-permute were compared to more common feature selection techniques such as LASSO, Elastic Net, and the f-test. The repeatability criteria features were determined by comparing the variability of radiomic features across repeat scans [17]. Unfortunately, classification models trained on this feature set consistently performed worse than those trained on the feature set that included all features, indicating that the repeatability criteria may have been too stringent and thus eliminated steatosis-relevant features. Averaged across all examined models, mRMR-permute was among the top feature selection techniques (with an MCC within 0.02 of the top feature selection technique) but had significantly fewer features compared to the other top performers, reducing the risk of overfitting and highlighting the importance of eliminating redundant radiomic features.

This study had a few notable limitations. The sample size (*n* = 42) was small relative to some machine learning studies but large compared to most nonhuman primate studies (median sample size: 5–10 [37]). A small sample size can limit the generalizability of machine learning models. For this reason, cross-validation and feature selection were used, whereas deep learning approaches such as convolutional neural networks were not used. This study was performed on crab-eating macaques and not humans, which limits the immediate clinical applicability of our results. However, crab-eating macaque and human livers share strong similarities in structure, function, and NAFLD pathogenesis [9,10,11,12,13], suggesting that our findings have a high likelihood of translating to humans. The use of macaques enabled us to correlate non-invasive imaging findings with extensive and detailed histopathological analyses across a larger area of the liver compared to core biopsies (typically collected in human studies). Because of the retrospective nature of our analysis, the macaques that received the high-fat diet were all males from a different geographical origin than the macaques (of both sexes) that received a standard diet. As a result, sex (X^2^ [1] = 12.0; *p_Bonferroni_* = 4.3 × 10^−3^) and country of origin (X^2^ [2] = 33.8; *p_Bonferroni_* = 3.7 × 10^−7^) were significantly different between the low- and high-steatosis-score groups. The dataset did not have enough power or diversity to determine the effects of these variables (and/or others) on the steatosis phenotype. Further, methods such as oversampling could not be used to correct the demographic imbalances because in many cases there were no samples to oversample (e.g., macaques from Cambodia that received the atherogenic diet were not available). In view of this limitation, we refrained from making statements or drawing conclusions related to the cause of steatosis and instead focused on predicting the histopathological feature of steatosis.

A bimodal distribution of steatosis severity was present in the dataset, possibly causing the increase in error in the mid-level steatosis scores (Figure 4b). The bimodal distribution also likely degraded the performance of the classification model. This skew in the distribution of steatosis severity is common in studies that contain a control group and a disease group but is not representative of the human population [5]. Although oversampling or generative approaches could be employed to overcome skewed data distributions, the best way to overcome this limitation and improve the model performance for mid-level steatosis scores would be to collect more data from macaques with mid-level steatosis scores. This could be accomplished by imaging macaques that receive 25:75, 50:50, and 75:25 ratios of normal and atherogenic chow. Other relevant histopathological hallmarks of liver disease (e.g., inflammation and necrosis) were sparsely represented in our dataset; therefore, radiomic signatures of these features could not be established in our study. Follow-up prospective imaging studies in animal models developing a wider range of histopathological features of liver disease will be needed to corroborate utility with respect to these histologic phenotypes.

In this study, we focused on classical machine learning and radiomics instead of deep learning approaches (e.g., convolutional neural networks). Classical machine learning models are more interpretable and robust when training data are limited compared to deep learning. Furthermore, by training classical machine learning models on mathematically defined radiomic features, we can explain both which imaging features are most important and what those features represent in an image.

## 5. Conclusions

Our study demonstrates that CT radiomics combined with classical machine learning offers a powerful, non-invasive approach for predicting histology-based hepatic steatosis scores. We showed that many CT radiomic features in the liver are redundant and compared feature selection methods to overcome this limitation. Of note, the mRMR-permute feature selection technique performed comparably to the top feature selection techniques with significantly fewer features. We also compared the performance of various machine learning models and analyzed the highest-performing model to determine the most important features for predicting histology-based hepatic steatosis scores. Approaches such as the one described in our study may ultimately enhance the diagnostic accuracy of CT imaging to detect hepatic steatosis and transform clinical practices by reducing the dependence on invasive biopsies, which are susceptible to sampling and visual assessment biases and are invasive procedures associated with patient discomfort and adverse effects.

## Figures and Tables

**Table 1:** Cohort description.

Metric	All Macaques	Low Steatosis (NAS_steatosis_ ≤ 1.5)	High Steatosis (NAS_steatosis_ > 1.5)	Statistic (df); *p*-Value; Cohen’s *d* [95% CI]
*n*	42	27	15	N/A
Diet [counts; non-atherogenic/atherogenic]	27/15	26/1	1/14	X^2^ (1) = 29.9; *p*_Bonferroni_ = 3.5 × 10^−7^
Age [yr; mean ± SD]	6.1 ± 1.7	5.8 ± 1.9	6.7 ± 1.1	Welch’s t (40) = 1.8; *p*_Bonferroni_ = 6.2 × 10^−1^; *d* = −0.2 [−0.8, 0.5]
Sex [counts; M/F]	26/16	11/16	15/0	X^2^ (1) = 12.0; *p*_Bonferroni_ = 4.3 × 10^−3^
Country of origin [counts; Mauritius/Cambodia/Indonesia]	15/6/21	1/6/20	14/0/1	X^2^ (2) = 33.8; *p*_Bonferroni_ = 3.7 × 10^−7^
History of SARS-CoV-2 exposure [counts; +/−]	30/12	18/9	12/3	X^2^ (1) = 0.3; *p*_Bonferroni_ = 1.0
NAS	1.5 ± 1.2	0.7 ± 0.5	3.0 ± 0.4	Welch’s t (32) = 16.6; *p*_Bonferroni_ = 2.8 × 10^−16^; *d* = 4.9 [3.7, 6.1]
NAS steatosis sub-score	1.3 ± 1.1	0.5 ± 0.4	2.6 ± 0.4	Welch’s t (31) = 15.9; *p*_Bonferroni_ = 1.2 × 10^−15^; *d* = 5.3 [4.0, 6.5]
NAS inflammation sub-score	0.2 ± 0.2	0.2 ± 0.2	0.4 ± 0.3	Welch’s t (24) = 2.6; *p*_Bonferroni_ = 1.4 × 10^−1^; *d* = 0.8 [0.2, 1.5]
NAS ballooning sub-score	0 ± 0	0 ± 0	0 ± 0	N/A

Means ± standard deviations and counts are given for quantitative and categorical descriptors, respectively. Welch’s *t*-tests and Chi-squared tests were used to test for differences between the binarized steatosis groups. The *p*-values were corrected for multiple comparisons using the Bonferroni correction. CI, confidence interval; df, degrees of freedom; M/F, male/female; NAFLD, nonalcoholic fatty liver disease; NAS, NAFLD activity score; NAS_steatosis_, NAS steatosis sub-score; SARS-CoV-2, severe acute respiratory syndrome coronavirus 2; SD, standard deviation.

## Data Availability

Due to the sensitive nature of nonhuman primate data and the relevant research collaboration agreements, we are not allowed to deposit the research data into an online public repository. However, the datasets generated and/or analyzed during the current study are available from the corresponding author upon reasonable request.

## Supplementary Materials

The following supporting information can be downloaded at https://www.mdpi.com/article/10.3390/diagnostics15182310/s1, Figure S1: Radiomic cluster heatmap; Figure S2: Radiomic correlation matrix; Table S1: Standard macaque diet details; Table S2: Radiomic feature list.

## Abbreviations

The following abbreviations are used in this manuscript:

AFLD	alcoholic fatty liver disease
AUC	area under the curve
BSL-4	biosafety level 4
CT	computed tomography
CV	cross-validation
GLCM	gray-level co-occurrence matrix
H&E	hematoxylin and eosin
HPC	High Performance Computing
HU	Hounsfield unit
kNN	k-nearest neighbors
LASSO	least absolute shrinkage and selection operator
MCC	Matthew’s correlation coefficient
mRMR	minimum redundancy, maximum relevance
NAFLD	nonalcoholic fatty liver disease
NAS	NAFLD activity score
NASH	nonalcoholic steatohepatitis
NIAID	National Institute of Allergy and Infectious Diseases
OCICB	Office of Cyber Infrastructure and Computational Biology
RMSE	root-mean-square error
ROC	receiver operating characteristic
ROI	region of interest
SARS-CoV-2	severe acute respiratory syndrome coronavirus 2
