# Supplementary material for: Computed Tomography Radiomics and Machine Learning for Prediction of Histology-Based Hepatic Steatosis Scores
Source: Diagnostics (Basel). 2025 Sep 11;15(18):2310. doi: 10.3390/diagnostics15182310 (PMC12468682; doi:10.3390/diagnostics15182310)
Supplement: Supplementary file 1 [file diagnostics-15-02310-s001.zip › diagnostics-3796929-supplementary.pdf]

Supplemental Material

Table of Contents

Figure S1 – Radiomic Cluster Heatmap 2
Figure S2 – Radiomic Correlation Matrix 3
Table S1 – Standard Macaque Diet Details 4
Table S2 – Radiomic Feature List 4

Figure S1 – Radiomic Cluster Heatmap

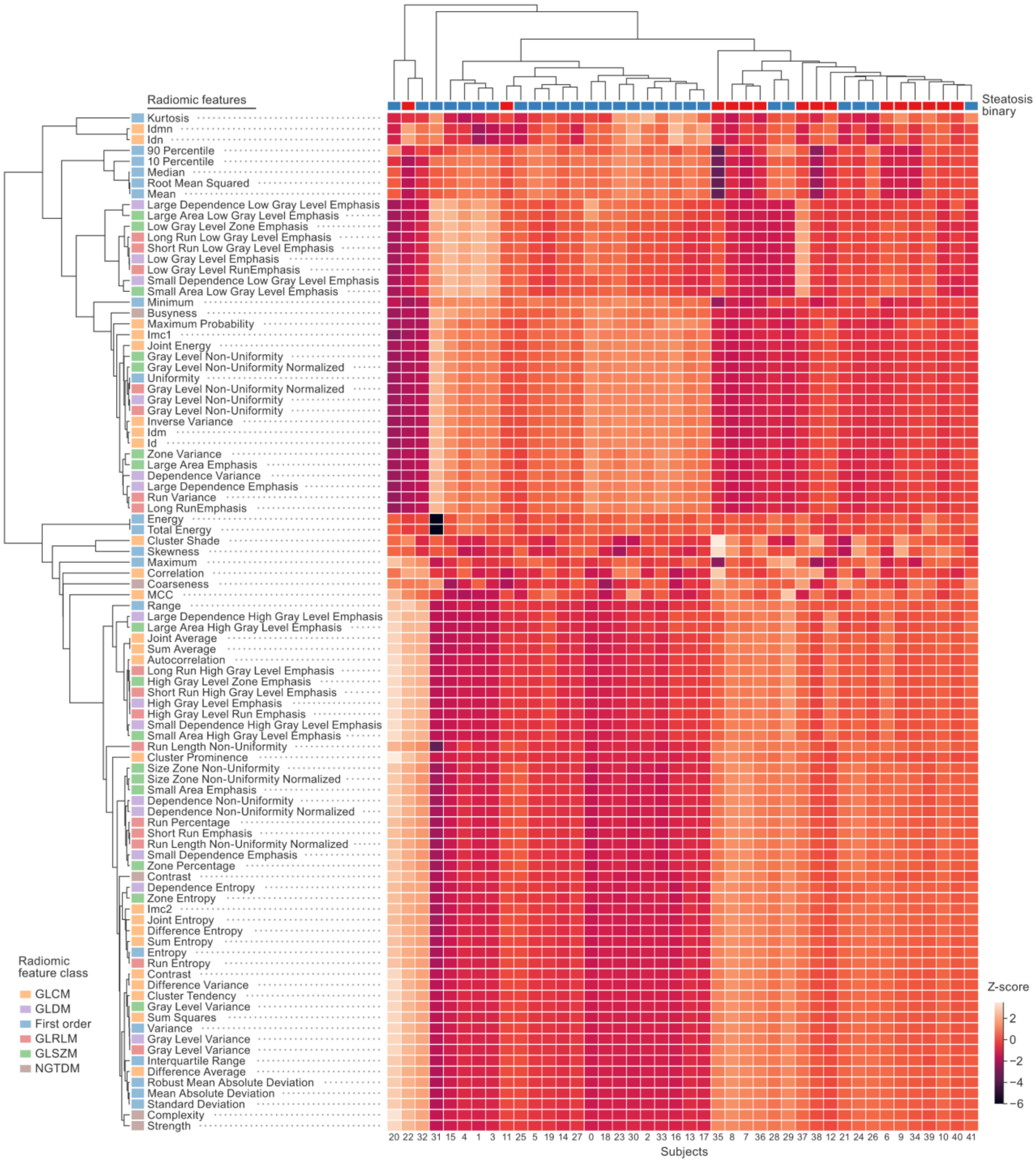

The hierarchical clustering heatmap illustrates the results of unsupervised clustering on the radiomic features and subjects. The rows are radiomic features and the columns are subjects. Heatmap values are the z-scores for particular subjects and features. The left-most column of colored boxes designates the class of the radiomic features and the top-most row designates the binarized steatosis group of the subject. The dendrograms designate the calculated clusters.

Figure S2 – Radiomic Correlation Matrix

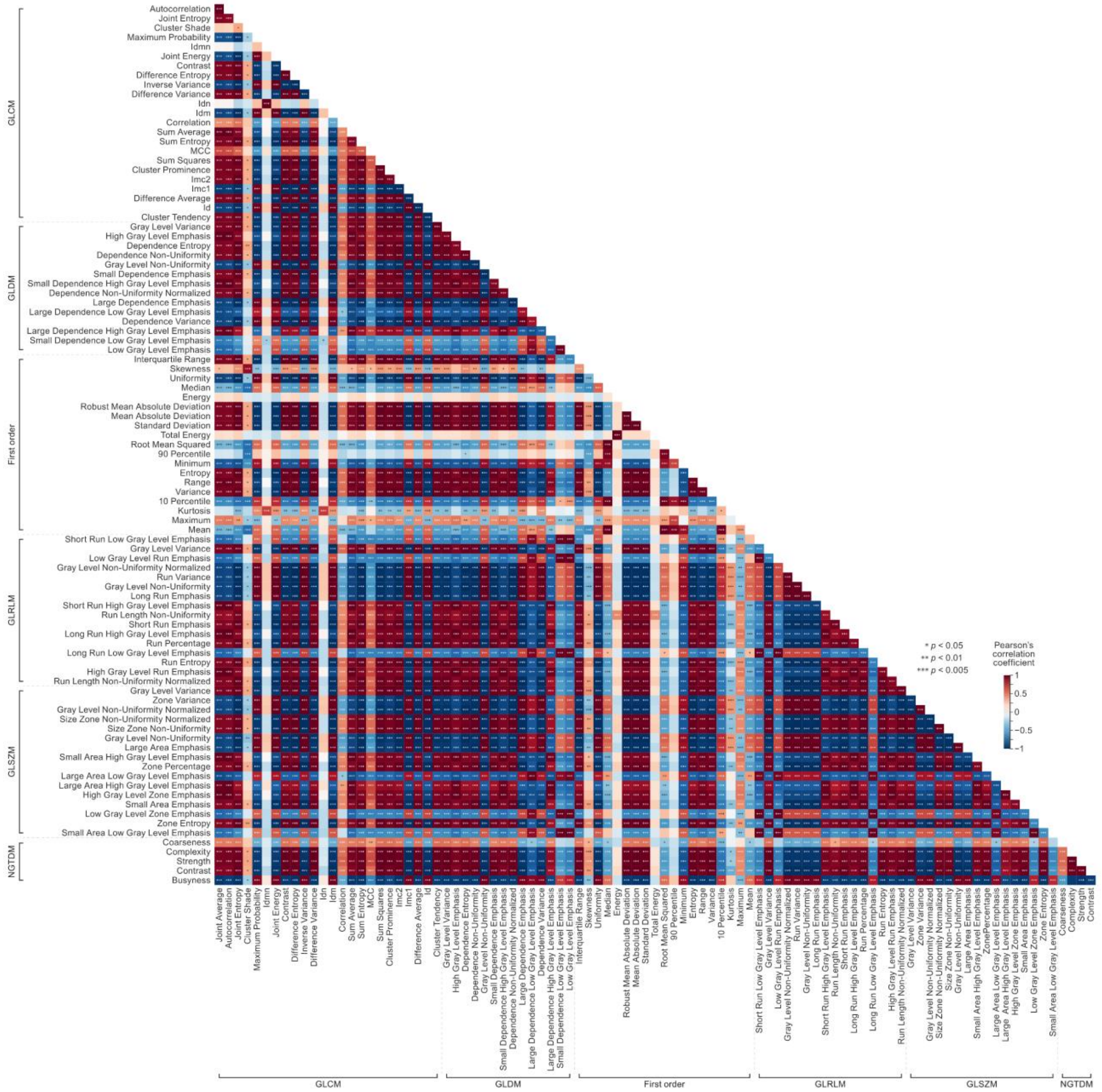

The correlation matrix shows correlations between radiomic features. Cells are colored according to Pearson's correlation coefficient and *p*-values are designated with asterisks. *$p < 0.05$, **$p < 0.01$, ***$p < 0.005$. This demonstrates that the large majority of radiomic features are highly correlated and likely redundant. The full resolution image can be accessed here: https://github.com/wtchu8/IRF_ML_Steatosis_pub/blob/main/supplementary_materials/radio-radiocorr_matrix.png

Table S1 – Standard Macaque Diet Details

Name	Link
LabDiet 5045	https://www.labdiet.com/product/detail/5045-high-protein-monkey-diet
LabDiet 5038	https://www.labdiet.com/product/detail/5038-monkey-diet
Teklad NIB Primate Diet 8773	https://insights.inotivco.com/hubfs/resources/data-sheets/8773.pdf

Table S2 – Radiomic Feature List

Group	Feature Name
First-order	10th percentile, 90th percentile, Energy, Entropy, Interquartile Range, Kurtosis, Maximum, Mean Absolute Deviation, Mean, Median, Minimum, Range, Robust Mean Absolute Deviation, Root Square Mean, Skewness, Total Energy, Uniformity, Standard Deviation, Variance
Gray Level Co-occurrence Matrix (GLCM; second-order)	Autocorrelation, Cluster Prominence, Cluster Shade, Cluster Tendency, Contrast, Correlation, Difference Average, Difference Entropy, Difference Variance, Inverse Difference, Inverse Difference Moment, Inverse Difference Moment Normalized, Inverse Difference Normalized, Informational Measure of Correlation 1, Informational Measure of Correlation 2, Inverse Variance, Joint Average, Joint Energy, Joint Entropy, Maximal Correlation Coefficient, Maximum Probability, Sum Average, Sum Entropy, Sum Squares
Gray Level Run Length Matrix (GLRLM; second-order)	Gray Level Non-Uniformity, Gray Level Non-Uniformity Normalized, Gray Level Variance, High Gray Level Run Emphasis, Long Run Emphasis, Long Run High Gray Level Emphasis, Long Run Low Gray Level Emphasis, Low Gray Level Run Emphasis, Run Entropy, Run Length Non-Uniformity, Run Length Non-Uniformity Normalized, Run Percentage, Run Variance, Short Run Emphasis, Short Run High Gray Level Emphasis, Short Run Low Gray Level Emphasis
Gray Level Size Zone Matrix (GLSZM; second-order)	Gray Level Non-Uniformity, Gray Level Non-Uniformity Normalized, Gray Level Variance, High Gray Level Zone Emphasis, Large Area Emphasis, Large Area High Gray Level Emphasis, Large Area Low Gray Level Emphasis, Low Gray Level Zone Emphasis, Size Zone Non-Uniformity, Size Zone Non-Uniformity Normalized, Small Area Emphasis, Small Area High Gray Level Emphasis, Small Area Low Gray Level Emphasis, Zone Entropy, Zone Percentage, Zone Variance

Neighboring Gray Tone Difference Matrix (NGTDM; second-order)	Busyness, Coarseness, Complexity, Contrast, Strength
Gray Level Dependence Matrix (GLDM; second-order)	Dependence Entropy, Dependence Non-Uniformity, Dependence Non-Uniformity Normalized, Dependence Variance, Gray Level Non-Uniformity, Gray Level Variance, High Gray Level Emphasis, Large Dependence Emphasis, Large Dependence High Gray Level Emphasis, Large Dependence Low Gray Level Emphasis, Low Gray Level Emphasis, Small Dependence Emphasis, Small Dependence High Gray Level Emphasis, Small Dependence Low Gray Level Emphasis
